# A novel *Tetrahymena thermophila* sterol C-22 desaturase belongs to the fatty acid hydroxylase/desaturase superfamily

**DOI:** 10.1016/j.jbc.2022.102397

**Published:** 2022-08-18

**Authors:** María L. Sanchez Granel, Nicolás G. Siburu, Annamária Fricska, Lucas L. Maldonado, Laura B. Gargiulo, Clara B. Nudel, Antonio D. Uttaro, Alejandro D. Nusblat

**Affiliations:** 1 Instituto de Nanobiotecnología (NANOBIOTEC), CONICET, Facultad de Farmacia y Bioquímica, Universidad de Buenos Aires, Buenos Aires, Argentina; 2 Instituto de Biología Molecular y Celular de Rosario, CONICET, Facultad de Ciencias Bioquímicas y Farmacéuticas, Universidad Nacional de Rosario, Rosario, Argentina; 3 Instituto de Investigaciones en Microbiología y Parasitología Médica (IMPaM), CONICET, Facultad de Medicina, Universidad de Buenos Aires, Buenos Aires, Argentina

**Keywords:** cholesterol, sterols, cytochrome P450, lipid, protein evolution, fatty acid, endoplasmic reticulum, ciliates, gene duplication, fatty acid desaturase family, CUB, codon usage bias, FAD, fatty acid desaturase family, FAHD, fatty acid hydroxylase/desaturase superfamily, HGT, horizontal gene transfer, NISE, Nonhomologous isofunctional enzymes

## Abstract

Sterols in eukaryotic cells play important roles in modulating membrane fluidity and in cell signaling and trafficking. During evolution, a combination of gene losses and acquisitions gave rise to an extraordinary diversity of sterols in different organisms. The sterol C-22 desaturase identified in plants and fungi as a cytochrome P-450 monooxygenase evolved from the first eukaryotic cytochrome P450 and was lost in many lineages. Although the ciliate *Tetrahymena thermophila* desaturates sterols at the C-22 position, no cytochrome P-450 orthologs are present in the genome. Here, we aim to identify the genes responsible for the desaturation as well as their probable origin. We used gene knockout and yeast heterologous expression approaches to identify two putative genes, retrieved from a previous transcriptomic analysis, as sterol C-22 desaturases. Furthermore, we demonstrate using bioinformatics and evolutionary analyses that both genes encode a novel type of sterol C-22 desaturase that belongs to the large fatty acid hydroxylase/desaturase superfamily and the genes originated by genetic duplication prior to functional diversification. These results stress the widespread existence of nonhomologous isofunctional enzymes among different lineages of the tree of life as well as the suitability for the use of *T. thermophila* as a valuable model to investigate the evolutionary process of large enzyme families.

Sterols are polycyclic triterpenoids found in most eukaryotic cells derived from the cyclization of oxidosqualene by different related cyclases. They have essential roles in modulating membrane fluidity and permeability, serving as precursors of bile salts and steroid hormones that play an important role in cell signaling and trafficking (1). It is assumed that the last eukaryotic common ancestor had the potential to make a wide array of different sterols and subsequent evolution over the eukaryotic tree occurred through tinkering *via* differential enzyme losses and specializations in the various eukaryotic lineages. The presence or absence of different enzymes such as cyclases, oxidases, desaturases, reductases, isomerases, transferases, among others, gave rise to an extraordinary diversity of sterols in different organisms (2). For example, a prominent structural difference of sterols in animals (cholesterol), most fungi (ergosterol), and plants (stigmasterol) lies in the presence or absence of the C22 double bond. The enzyme responsible for the C22 desaturation reaction has been identified in fungi and plants and belongs to the cytochrome P-450 monooxygenase superfamily (3, 4). Fungal CYP61 is involved in the ergosterol biosynthetic pathway, desaturating at C22(23) the precursor ergosta-5,7,24(28)-trienol in the penultimate step. In the case of the phytosterol biosynthetic pathways in plants, CYP710 is responsible for the desaturation at C22(23) of the precursors for stigmasterol, brassicasterol, or crinosterol in the last step. It was suggested that CYP61 and CYP710 have evolved from a duplication of cytochrome CYP51, which is considered the first eukaryotic cytochrome P-450, before the separation of the ancestor of the Viridiplantae. This explains the finding of the sterol C-22 desaturase activities that are widespread in Viridiplantae, fungi, choanoflagellates (close unicellular relatives of animals), and in early-branching animals. Later during animal evolution, the loss of this enzyme led cholesterol being the predominant sterol (5).

Among the different organisms that desaturate at the C22(23) position of the sterol structure is the free-living unicellular ciliate *Tetrahymena* (6). Interestingly, instead of harboring sterols in its membranes, the ciliate produces tetrahymanol, a pentacyclic triterpenoid that can be synthesized in the absence of molecular oxygen (7). However, in the presence of sterols in the growth medium, *Tetrahymena* suppresses the synthesis of tetrahymanol and replaces it by the acquired sterol, directly or previously modified, into their membranes (8). These modifications include desaturations at the C5(6), C7(8), and C22(23) positions in the sterol structure and the removal of the C-24 ethyl group from C-29 sterols in the case of phytosterols (Fig. 1) (9). Protein-coding genes of the C-5 desaturase (*DES*5A), the C-7 desaturase (*DES*7), and the C-24 de-ethylase (*DES*24) were recently identified by using reverse genetic approaches in the ciliate *Tetrahymena thermophila* (10, 11, 12). These proteins are localized in the endoplasmic reticulum (ER) and, in the case of the sterol C-5 desaturase and C-24 de-ethylase, belong to the short-spaced subfamily of the fatty acid hydroxylase/desaturase superfamily (FAHD). Two FAHD subfamilies can be established based on the length of the spacing between the first and third histidine motifs: the short (fatty acid hydroxylase family) and the long-spaced (fatty acid desaturase family, FAD) (13). The C-7 sterol desaturase, on the other hand, belongs to the Rieske-type nonheme iron oxygenases (12). The identification of the sterol C-22 desaturase has been difficult, because orthologs of the P-450 oxygenases belonging to the family of sterol C-22 desaturase are not present in the *T. thermophila* genome (14). Furthermore, the biochemical characterization of this enzyme in the ciliate (a requirement for cytochrome *b*_5_ and lack of inhibitory effect by azoles) (15) showed to be different from the sterol C-22 desaturases characterized in yeast and plants (4, 16, 17), suggesting that the C-22 desaturation in *Tetrahymena* might be catalyzed by an enzyme unrelated to P-450 oxygenases.

**Figure 1:**
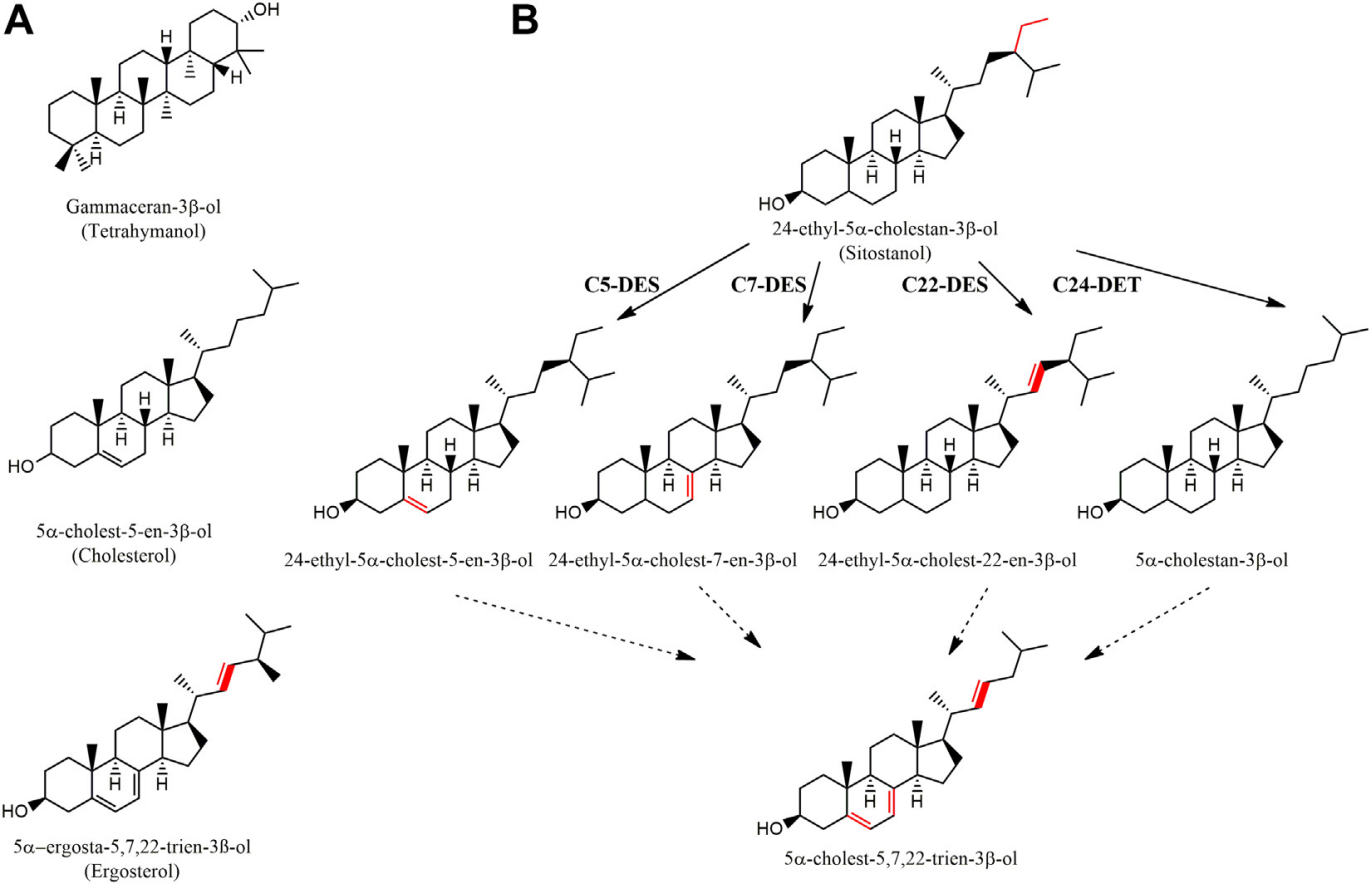
***Tetrahymena thermophila*****sterol metabolism.***A*, tetrahymanol, cholesterol and ergosterol structures shown for comparison. *B*, pathway of sterol metabolism in *Tetrahymena thermophila*. In *red* are shown the sterol modifications. Δ^22^ double bond is shown in *bold red*. C-5 DES, sterol C-5 desaturase; C-7 DES, sterol C-7 desaturase; C-22 DES, sterol C-22 desaturase; C-24 DET, sterol C-24 deethylase.

Enzymes that lack detectable sequence similarity but catalyze the same biochemical reactions are referred to as nonhomologous isofunctional enzymes (NISEs) (18). These enzymes have independent evolutionary origins; therefore, they have distinct structural folds. Several studies have suggested that the fraction of Enzyme Commission (EC) nodes (individual biochemical reactions) that include two or more proteins without detectable sequence similarity to each other, accounts for a substantial 10% among enzymes included in the EC system (19, 20, 21). In general, the diversity related to the classes of enzymes, biochemical pathways, protein folds, and phylogenetic lineages shows an irregular distribution of NISE. These enzymes seem to be originated from preexisting enzymes with related activities and specificities, typically enzymes within a superfamily, following duplication or horizontal gene transfer (HGT) (22).

The sterol C-22 desaturase was characterized for the first time as a cytochrome P-450 superfamily member in the yeast *Saccharomyces cerevisiae*. The enzyme was purified using an approach based on the spectral properties of cytochrome P-450 and reconstituting the activity *in vitro* with phospholipids and P-450-reductases (23). Soon after, the coding gene was identified by complementation of a *S. cerevisiae* random mutant using a negative selection protocol involving screening for nystatin-sensitive transformants (3). Subsequently, many sterol desaturases have been identified in other fungi and plants by the search of similar nucleotide or amino acid sequences. In *T. thermophila*, the sterol C-22 desaturase activity was shown to be induced by the presence of sterols in the culture media (15). Based on this feature, in a previous work, we identified putative sequences using a transcriptional analysis of the ciliate exposed to exogenous cholesterol (24). In this work, we confirmed and identified two paralogous genes coding for two proteins with sterol C-22 desaturase activity belonging to a different enzyme family, the FAD. Furthermore, we studied its possible evolutionary origin and the mechanism underlying this process.

## Results

### Identification and functional analyses of sterol C-22 desaturase from T. thermophila

Blast analysis using reviewed (Swiss-Prot) C22-desaturases from plants and fungi as queries identified only weak putative sequences (E value higher than 1.0E^−15^) in the *T. thermophila* genome. None of the two motifs, substrate recognition sites 1 and 3 (NX_5_GX_2_HX_3_RX_6_FTX_3_ALXY and FD/TFLFAA/SQDAS/TT/SS), considered the signature of sterol C-22 desaturases, were found in those sequences (5).

Consequently, other strategies had to be tested for the identification of the coding gene. As noticed during the biochemical characterization of *T. thermophila* sterol C-22 desaturase activity in microsomes, the presence of sterols in the culture media increased the yield of recovery of the enzyme, thus highlighting the importance of inducible mechanisms for enzyme expression. A differential genome-wide transcriptomic analysis of the ciliate, growing in the absence or presence of cholesterol, detected numerous upregulated genes by the sterol. Interestingly, the second and third most upregulated genes code for oxygenases, which were selected as putative sterol desaturases (*DES22A* and *DES22B*) (24).

**Figure 2:**
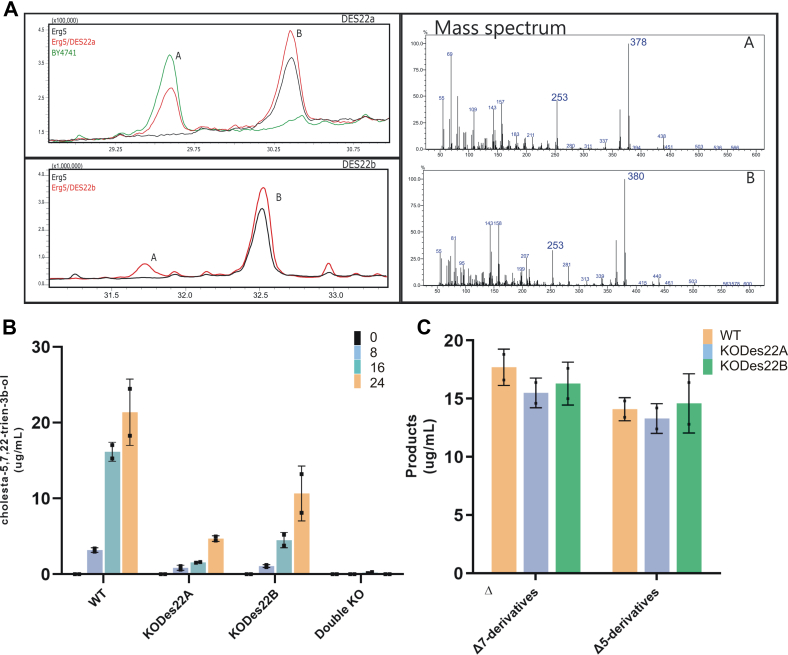
**Functional characterization of*****T. thermophila*****C22 sterol desaturases.***A,* GC-MS analysis of sterol extracts are shown from a C22 mutant yeast strain (erg5), which accumulates 5,7-ergostadien-3β-ol (B) rather than ergosterol (5,7,22-ergostatrien-3β-ol) (A); from erg5 transformed with *DES22*A (rDes22A); from erg5 transformed with *DES22*B (rDES22B) and from a wildtype yeast strain (BY4741). A and B refer to product and substrate, respectively. *B*, conversion products of *T. thermophila* strains at different hours. HPLC analysis of sterols extract from the WT, simple KO (KODes22A KODes22B), and double KO strains with medium supplemented with 5,7-cholestadien-3β-ol. *C*, product formed from the WT and simple KO (KODes22A and KODes22B) strains with medium supplemented with cholesterol (Δ5 derivative products) or lathosterol (Δ7 derivative products).

Both genes belong to a group of four paralogs in the ciliate with 59.1% identity in their amino acid sequences. In order to evaluate whether *putDES22A* and *putDES22B* (TTHERM_00129290 and TTHERM_00085010, respectively) display sterol C-22 desaturase activity, we used a heterologous expression system with a C22-desaturase null mutant *S. cerevisiae* strain (*erg5*). This strain accumulates 5,7-ergostadien-3β-ol rather than ergosterol (5,7,22-ergostatrien-3β-ol) as the major sterol. As shown in Figure 2*A*, both *erg5* yeast mutant strains expressing the *T. thermophila putDES22A* or *putDES22B* genes exhibited sterol C-22 desaturase activity, enabling the biosynthesis of ergosterol.

**Figure 3:**
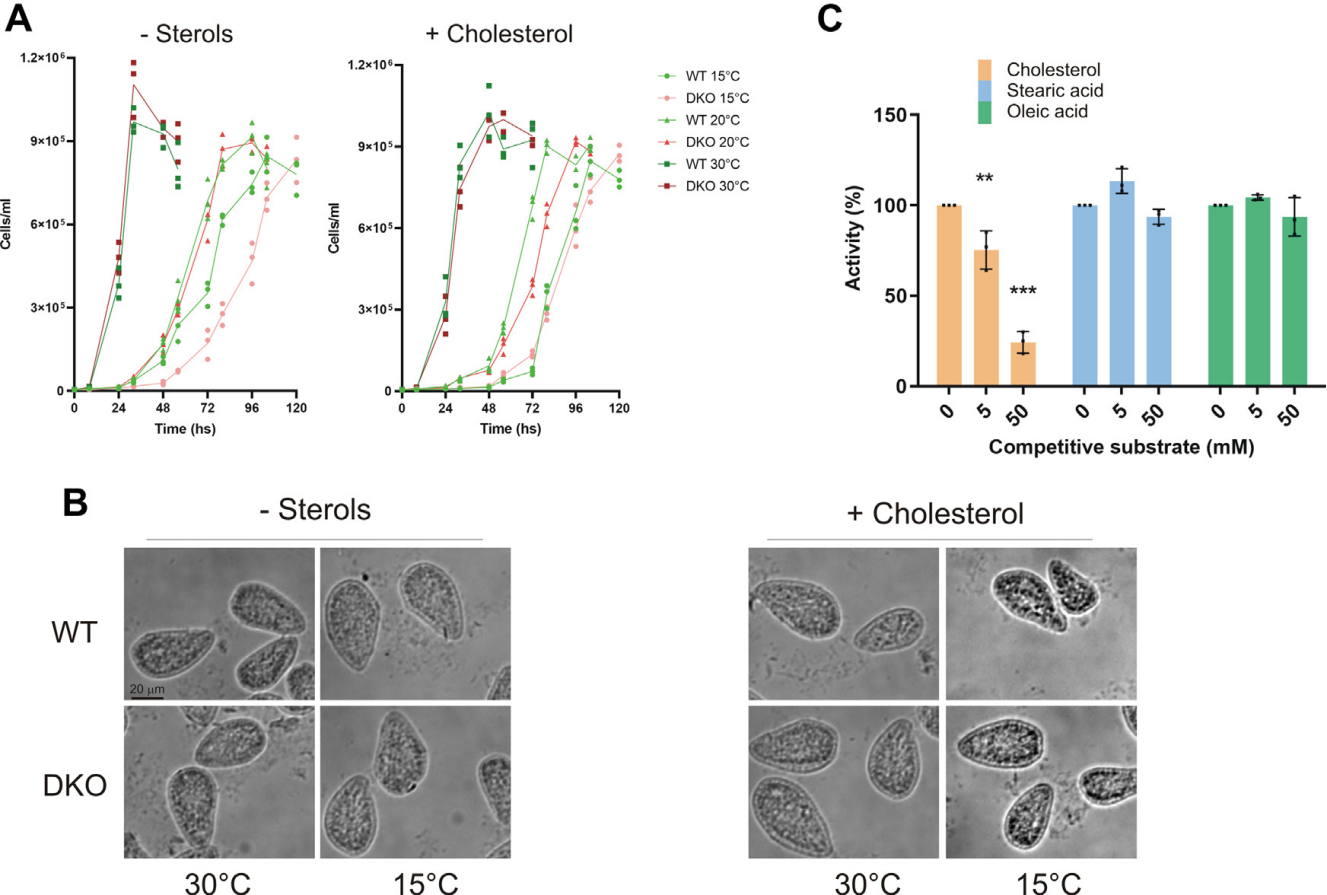
**Physiological effects of WT and C22 KO mutant strains.***A*, growth of WT and double KO (DKO) strains measured at 15 °C, 20 °C, and 30 °C in SPP medium with cholesterol and without any sterol. Results are shown as individual replicates (n = 3) with means connected. *B*, cellular morphology of WT and double KO *T. thermophila* strains. *C*, competition assay of C22 sterol desaturase activity by fatty acids. The assay was performed on 2 ml of microsomal fraction. The activity was calculated as a fraction of the initial activity and expressed in (%). Results are shown as the mean ± SD (n = 3). *Asterisks* denote statistical significances (∗∗∗*p* ≤ 0.001; ∗∗*p* ≤ 0.01) in comparison to the point without competitive substrate. Two-way ANOVA followed by Bonferroni's multiple comparison test was used to assess differences.

To further confirm conclusively that both genes codify for a sterol C-22 desaturase, we generated *T. thermophila* single KO mutant strains (KODes22A and KODes22B), as well as a double KO mutant strain (KODes22AB) by eliminating the coding genes. In the case of the single mutants, a traditional somatic knockout procedure through homologous recombination in the macronucleus genome was performed, whereas in the double KO mutant strain, two methods were combined. The *DES22A* gene was eliminated by a targeted ectopic DNA elimination (co-deletion) procedure and the *DES22B* gene by a somatic knockout procedure. Comparative analysis of the conversion products formed by the WT, single KO, and double KO mutant strains with medium supplemented with pro-vitamin D_3_ (5,7-cholestadien-3β-ol) showed that sterol C-22 desaturase activity was significantly decreased in both single KO mutant strains, whereas in the double KO strain, no sterols with a double bond at the C-22 position were detected (Fig. 2*B*). Abolishing the C22 desaturase did not affect other sterol desaturase activities (*i.e.*, C7 and C5 sterol desaturases) in any KO strain. In fact, both single KO mutant strains showed similar C-7 and C-5 sterol desaturase activities upon supplementation of the culture medium either with cholesterol (5-cholesten-3β-ol) or with lathosterol (7-cholesten-3β-ol), thus indicating that these desaturases were not impaired (Fig. 2*C*). The disruption of the DES22A and DES22B genes in the mutant strains have no other evident physiological effects on the organism. No significant differences in the growth of double KO and WT at different temperatures with and without sterols were recorded (doubling time and total biomass yield, *t* test *p* < 0.05). Fatty acid content, cellular behavior, cellular morphology, and movement did not show neither differences (Table S3 and Fig. 3, *A* and *B*). To get more information about the substrate selectivity, we performed competitive assays between cholesterol and fatty acids in *in vitro* experiments. Figure 3*C* shows that the sterol C22 desaturase activity over the cholesterol is not affected by the addition of stearic and oleic.

**Figure 4:**
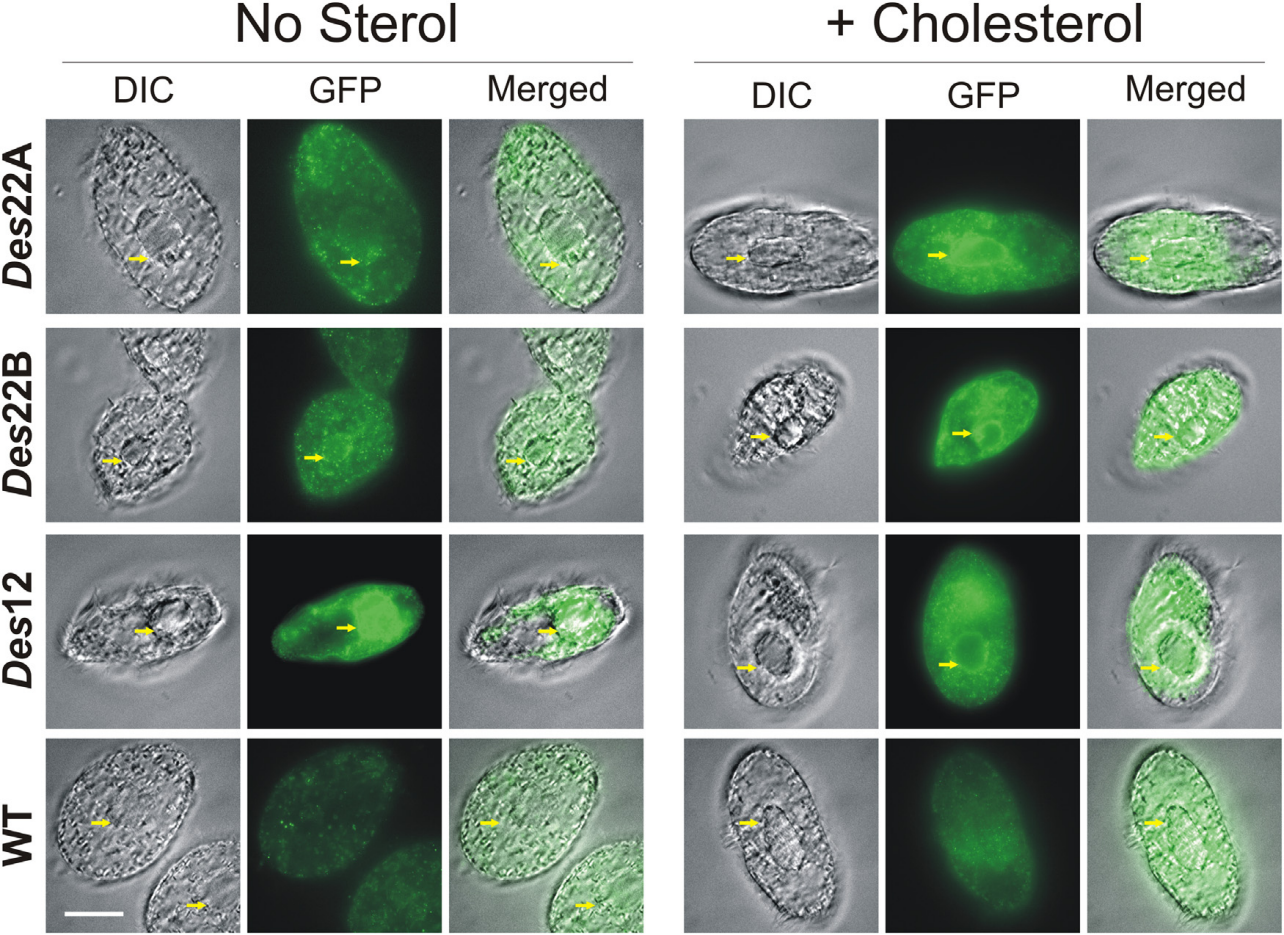
**Localization of GFP-tagged *DES22*A, *DES22*B, and *DES*12 (Δ12 fatty acid desaturase) in *T. thermophila*.** Immunofluorescence of GFP-tagged desaturases grown in SPP medium and in SPP medium supplemented with cholesterol. The GFP was localized by anti-GFP primary antibody followed by labeling with anti-rabbit-Alexa Fluor 488 secondary antibody. The *yellow arrow* shows the macronucleus. Scale bar in white: 20 mm.

In order to localize the enzyme in *T. thermophila*, we tagged the Des22Ap and Des22Bp in different strains at the C terminus with EGFP. To reduce potential misexpression artefacts in the ciliate, we targeted the tagged gene to its endogenous locus. Due to the high autofluorescence background even in WT cells, we increased the selectivity of the signal using anti-GFP antibody and indirect immunofluorescence microscopy. Microscopic analysis of both Des22A-GFP and Des22B-GFP strains showed a labeling around the nucleus after supplementation with cholesterol for 3 h. This perinuclear pattern is consistent with an ER localization of the fatty acid Δ12 desaturase from *Tetrahymena tetrahymena* used as control (Fig. 4). The ER localization is also in concordance with the results of the *in silico* prediction of protein subcellular localization (Euk-mPLoc 2.0) (25).

### Des22Ap and Des22Bp belong to the FAHD

**Figure 5:**
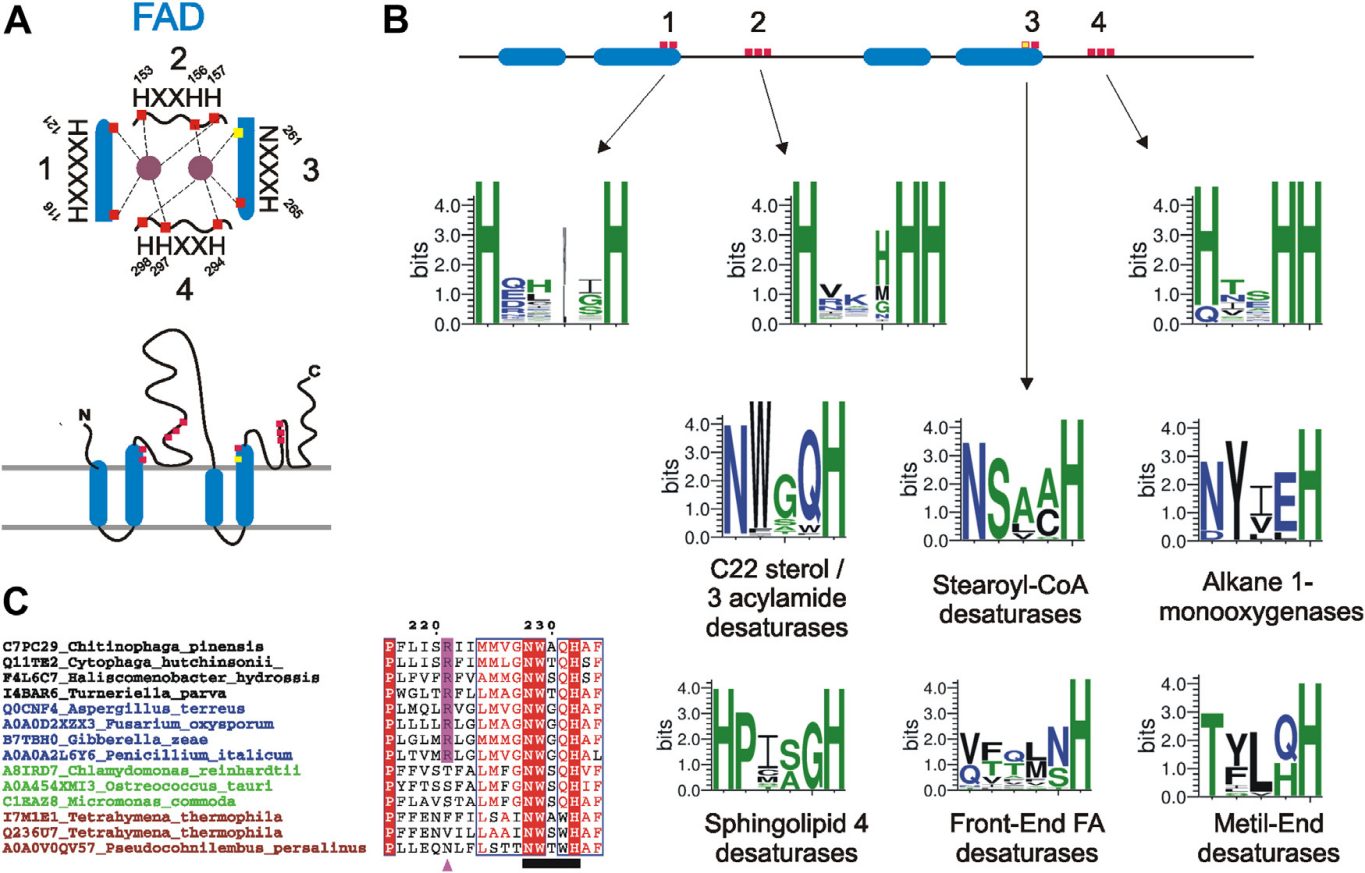
**Fatty acid desaturase family.***A*, schematic representation of the dimetal center from the stearoyl-CoA desaturase (Uniprot ID: P13516) (up) and the topology diagram (down). The coordinating His residues are shown in *red*, and the Asn residue of the conserved motifs in *yellow*. Transmembrane domains are shown with a *thick blue line*. N and C termini are located in the outer side of the endoplasmic reticulum membrane. The representation is a reproduction of Figure 1 of (51). *B*, conserved histidine motifs of the families belonging to the FAD family. The first, second, and fourth motifs are conserved among all the FAD families, whereas the third is specific to each family. Sequence logos were generated with WebLogo (76) using sequences listed in Table S2. The sequence logo of the FAD family was generated with the sum of each individual FAD family sequences. *C*, multiple sequence alignment of the C22 sterol/acylamide-delta-3(E)-desaturase family sequences from bacteria (*black*), fungus (*blue*), viridiplanteae (*green*), and ciliates (*red*), showing the conserved Arg249 (*purple triangle*) of the mammalian stearoyl-CoA desaturase involved in the stabilization of the transmembrane helices. The *black bar* shows the third conserved histidine motif. I7M1E1 is the Des22Ap and Q236U7 the Des22Bp. FAD, fatty acid desaturase family.

*DES22A* and *DES22B* were the only two genes from a group of four paralogs in *T. thermophila* that showed to be expressed in any condition of the transcriptome analysis (with or without cholesterol). The two proteins have an identity of 50.6% and a similarity of 65.7%. To classify them into protein families, we searched in the InterPro resource, which uses predictive models provided by several different databases (26). No predicted protein family memberships were retrieved in either case. However, the domain architecture was assigned to a fatty acid desaturase for both enzymes. Proteins with these domains belong to the long-spaced subfamily of the FAHD superfamily (FAD), which are non-heme di-iron, integral membrane enzymes containing characteristic histidine motifs (27) (Fig. 5*A*). Blast and orthology analysis using Des22Ap and Des22Bp as queries retrieved only one sequence that was experimentally studied. Interestingly, the sequence A0A2H3GTI9 from fungus *Gibberella zeae* (*Fusarium graminearum*) has acylamide-delta-3(E)-desaturase activity (28). So far, none of the reports on lipid composition in *Tetrahymena*, in several growth conditions and also from various research teams including ours, have revealed the presence of any delta-3 unsaturated compound in any lipid analysis, neither as free fatty acids nor in the fatty acyl moiety of sphingolipids or triglycerides, generating uncertainty as to the real activity of the expressed genes in the ciliate.

The stearoyl-CoA desaturase family has four histidine motifs that are involved in the coordination of the di-iron catalytic center. Analysis of the histidine motifs of the other families belonging to the FAD shows that three of them (first, second, and fourth) are highly conserved, whereas one (the third motif) is specific to each enzyme group (Fig. 5*B*). The third motif belonging to the sterol C-22/acylamide-delta-3(E)- desaturases, as well as the stearoyl-CoA desaturases and the alkane 1-monooxygenase enzyme families, displays a conserved asparagine that is involved in the coordination of the dimetal center through a hydrogen-bonded water molecule (29). Another feature of this enzyme family is the presence or absence of a conserved arginine (Arg249 in mammalian stearoyl-CoA desaturase) which help to stabilize the transmembrane helices (29). Interestingly, the arginine is present in desaturases from bacteria and fungus and absent in desaturases from Viridiplanteae and ciliates (Fig. 5*C*). These differences could be related to the substrate preferences of the enzymes (sphingolipids in fungus *versus* sterols in ciliates). However, experimental analyses are needed to confirm it. A prediction analysis suggests that Des22p are composed of six or five transmembrane helices and 17 or 20 α-helices (Des22Ap and Des22Bp respectively) (Fig. S1). This theoretical protein structure differs significantly from the resolved structure of a mammalian stearoyl-CoA desaturase, which is composed of four transmembrane helices and 11 α-helices (29).

### Origin of the sterol C-22 desaturase family

**Figure 6:**
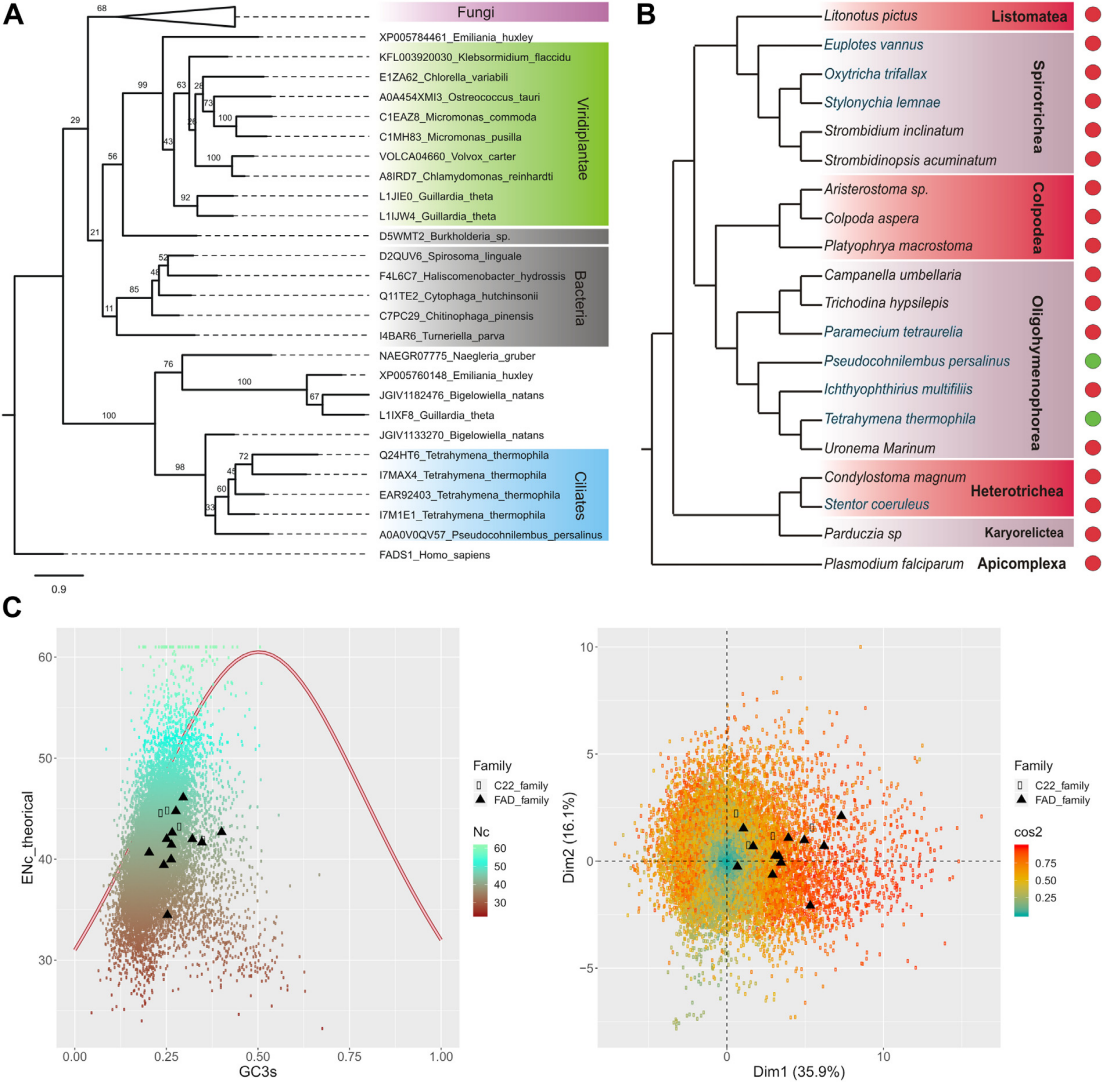
**Phylogenetics and HGT analysis of C22 sterol desaturases.***A*, phylogenetic analysis of C22 sterol desaturase. I7M1E1 is the Des22Ap and Q236U7 the Des22Bp. For phylogenetic analyses, the MAFFT program with the E-INS-i iterative refinement method was used to obtain the multiple alignment from 90 protein sequences listed in Table S2. This multiple sequence alignment (MSA) was used to construct the phylogeny by the maximum likelihood method using the PhyML 3.0 software optimized by subtree pruning and regrafting and nearest neighbour interchange and a support of 100 bootstraps (69). The model LG+G+I for the MSA was selected using the smart model selection method (70). Fungi sequences were collapsed for a better visualization of the phylogenetic tree. The noncollapsed tree, showing sequences from different fungi phyla, is shown in Fig. S4. *B*, scheme of the phylum Ciliophora based on the phylogenetic analyses from (77, 78, 79, 80). *Green ovals* denote the presence of C22 sterol desaturase orthologs and red ovals the absence. *C*, distribution of the effective number of codons (ENc) in relation to the GC3 content (GC content of synonymous codons in the third position) of *T. thermophila* genes (*left panel*). The *solid line* represents the expected curve when codon usage bias is only affected by mutation pressure (up). Principal component analysis of relative synonymous codon usage in *T. thermophila* (*right panel*). The axis shows the percentage accounting for the total RSCU variation (down). *Black circles and triangles* indicate genes belonging to the C22 sterol desaturase family and to other members of the fatty acid desaturase family respectively. HGT, horizontal gene transfer.

The phylogenetic analysis of sterol C-22/acylamide-delta-3(E) desaturase sequences grouped both *T. thermophila DES*22 genes with the other two *T. thermophila* paralogs and with other sequences of the ciliate *Pseudocohnilembus persalinus* into a monophyletic branch (Fig. 6*A*). Interestingly, coding sequences were retrieved only in these two genera of the ten ciliates with complete sequenced genomes that were investigated (Fig. 6*B*). The tree also shows a large monophyletic branch composed of different sequences from fungal phyla which contain the acylamide-delta-3(E)-desaturase from a small monophyletic branch of green algae sequences (Fig. S2), a small number of bacterial sequences from different phyla and a branch composed of few sequences from unrelated groups (*Emiliania huxley*; Haptista; *Naegleria gruber*: Discoba; *Guillardia theta*: Cryptista; and *Bigelowiella natans*: Rhizaria) (30). The patchy phylogenetic distribution as well as the absence of eukaryotic sequences from other groups of the tree of life such as stramenopiles, CRuMS, amoebozoans, animals and land plants suggest as a possible scenario that different branches could be originated *via* HGT. Another possible scenario for the origin of sterol C22 desaturases in ciliates may be by the duplication of preexisting enzymes that take on a new cellular function following changes to the substrate binding site or catalytic mechanism. To discern between these two scenarios, we analyzed possible HGT events by parametric methods. One of the most widely used and most efficient methods for detecting HGT are those that rely on codon usage bias (CUB) (31, 32). The ENc-GC3s plot and PCA analysis (Fig. 6*C*) showed that the CUB of the four sequences belonging to the sterol C22 desaturases in *T. thermophila* are simply a reflection of the overall AT rich codon usage in the ciliate genome and do not present different compositional characteristics from the rest of the FAD families of the ciliates. The same results were obtained from *Tetrahymena borealis*, *Tetrahymena elliotti*, and *Tetrahymena malacensis* (Fig. S3). Indeed, ENc values ranged from 36.46 to 46.15 and from 34.34 to 46.24 for C-22 desaturases and FAD genes, respectively, in the different species, thus minimizing the probability that an HGT event would be the origin of the sterol C22 desaturase family in *Tetrahymena*. Analysis of codon usage is also widely used to infer if genes are under mutation or selection pressure. The ENc-GC3s plot showed that in the *Tetrahymena* species, all the FAD genes were distributed below the expected curve (no selection pressure) showing ENc values <50. Indeed, the ∼98% value/percentage of the genome genes for all the species showed ENc values lower than 50, suggesting that the codon usage of most of the *Tetrahymena* genes is influenced by other factors in addition to the mutational bias. Other CUB analyses such as the neutrality plot and the PR2 bias plot demonstrated that selection pressure is the main evolutionary force that shapes the CUB in sterol C22 desaturases (Fig. S4).

**Figure 7:**
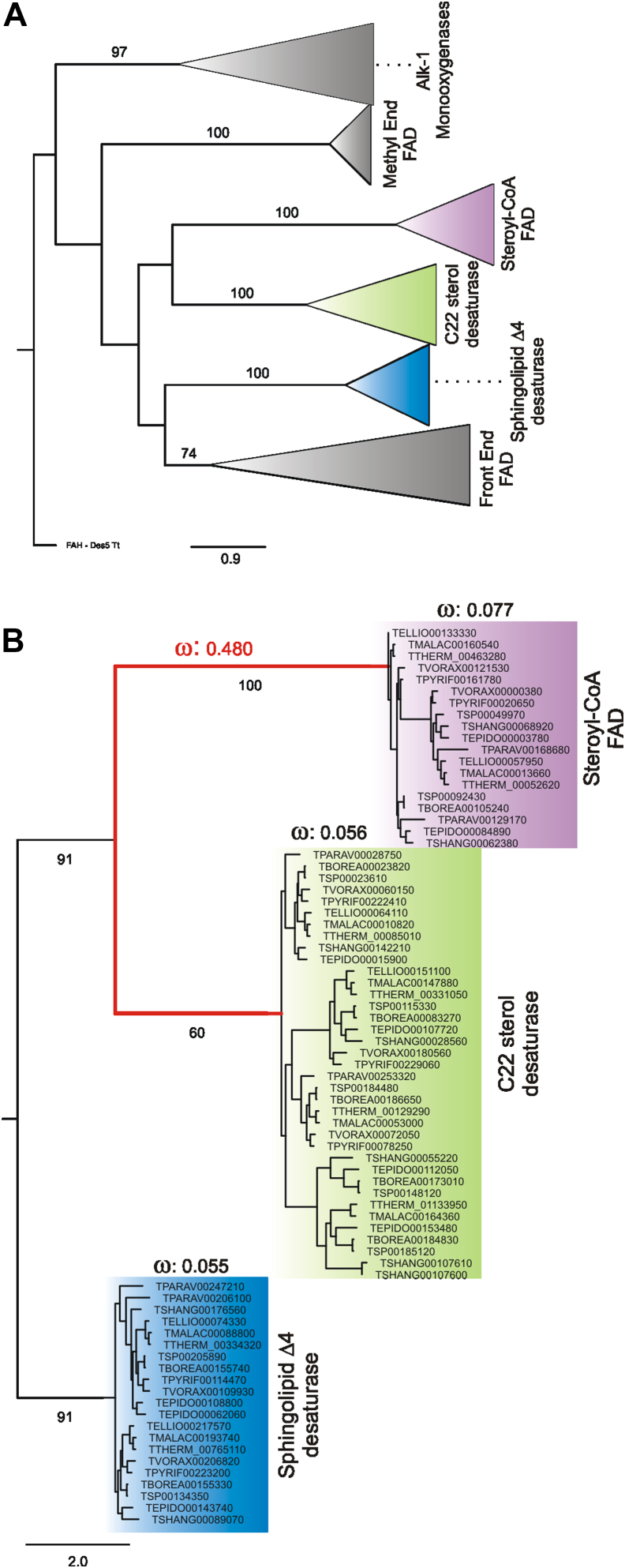
**Evolutionary origin of C-22 desaturases.***A*, phylogenetic analysis of proteins belonging to the fatty acid desaturases family from different ciliate species. For the phylogenetic analysis, 160 sequences listed in Table S2 were aligned using the MAFFT program with the E-INS-i Iterative refinement method. This multiple sequence alignment (MSA) was used to construct the phylogeny by the maximum-likelihood method using the PhyML 3.0 software optimized by subtree pruning and regrafting and nearest neighbour interchange and a support of 100 bootstraps. The model LG+G+I+F for the MSA was selected using the smart model selection method. Only bootstrap values higher than 50% are shown. The FAD families were classified according to the location of *T. thermophila* sequences previously classified in Cid, 2017. FAD families were collapsed for a better visualization of the phylogenetic tree. *B*, phylogenetic tree of 77 *Tetrahymena* CDS sequences of C22 sterol and stearoyl-CoA desaturase families using the sphingolipid Δ4 desaturase family to root the tree. The branch that predates the possible duplication event is shown in *red*. The selective pressure in different branches is shown as the ratio of substitution rates at nonsynonymous and synonymous sites, dN/dS (w), calculated with the CODEML analysis from the PAML package, using free-ratios (model = 2) of the branch model (71, 72). FAD, fatty acid desaturase family.

Subsequently, we analyzed the selective pressure in different branches of the FAD subfamily to infer if functional divergence after gene duplication could be the cause of the evolutionary origin of C-22 desaturases. After gene duplication, there is often a relaxed functional constraint that adapts the proteins to a new function, followed by a purifying selection that maintains the new functions (33). As shown in the phylogenetic analysis of proteins belonging to the FAD from different ciliate species (Fig. 7*A*), the C-22 desaturase seems to be originated by duplication from stearoyl-CoA desaturase family sequences; therefore, we estimated the selective pressures at different times in the phylogenetic tree using different species of *Tetrahymena*. The omega (ratio of substitution rates at nonsynonymous and synonymous sites, dN/dS) calculated for the branch of sterol C-22, stearoyl-CoA, and sphingolipid Δ4 desaturases (used as outgroup) was 0.056, 0.077, and 0.055, respectively, showing a high purifying selection over these three branches (Fig. 6*B*). By contrast, the branches immediately following the duplication event showed a significant increase in the evolutionary rate, omega: 0.480, probably due to the relaxation of selective constraints. These results, together with the parametric analysis, indicate a gene duplication which gave rise to a functional divergence as the most plausible scenario for the origin of the C-22 desaturase.

## Discussion

To date, three sterol desaturases that belong to different EC nodes have been identified: C-5, C-7 and C-22 desaturases (23, 34, 35). Although the three enzymes are integral membrane monooxygenases with apparent similarity to the reaction that they catalyze, the three enzymes do not share phylogenetic patterns, functional domains, topological motifs, nor electron transfer chains (ETCs). Using reverse genetic and bioinformatic approaches, the present study demonstrates that both *DES22A* and *DES22B* genes encode a novel type of sterol C22 desaturase in the ciliate *T. thermophila* that belongs to the long-spaced subfamily of the FAHD superfamily (FAD), therefore identifying a new class of NISE enzymes. The presence of analogous enzymes in the FAD subfamily has been previously documented for the alkane ω-hydroxylase which performs the same activity (catabolism of medium chain–length alkanes) as the cytochrome P450 alkane hydroxylase of the Cyp153 family (36, 37). Our findings add more evidence to the high catalytic plasticity of the FAHD superfamily already documented (13). They also expand the knowledge on the use of different substrates in the FAD family, adding sterols to the list of fatty acids (fatty acid desaturases), sphingolipids (sphingolipid Δ4 desaturase), and alkanes (alkane ω-hydroxylase). Interestingly, the phylogenetic analysis showed the presence of orthologous genes in fungal genomes that codify for proteins with another enzymatic activity, the acylamide-delta-3(E)-desaturase activity (28) showing also a high catalytic plasticity not only at a superfamily level but also at a protein family level. This feature has been reported many times for different enzymes that belong to different FAHD families (38, 39, 40), contributing to the evolution of the chemical diversity in nature.

Our bioinformatic analysis of the different enzymes from the FAHD superfamily enabled the identification of an extra histidine cluster that was only described in the stearoyl-CoA desaturase family (29), thus increasing the number of clusters from three (27) to four in each family necessary for the heme coordination in the active site. The analysis of C22 sterol/acylamide-delta-3(E) desaturase family also shows a conserved arginine in the sequences from bacteria and fungus not present in ciliates and Viridiplantae that could explain, at least partially, the different substrate preferences of the two enzymes classes (4, 41). Although single amino acid substitution has shown to change the substrate specificity for different fatty acids in FAHD (42), the change of specificity between two highly different substrates (sterols *versus* sphingolipids) would surely imply several substitutions as well as structural changes in the enzymes, not yet described. Our analysis showed no activity C-22 desaturase with fatty acids (stearic and oleic acid) suggesting a specificity for cholesterol and other sterols (43). Both C-22 desaturases appear to have similar specificities for cholesterol and lathosterol, with activities that may vary depending on their gene expression. These results are in concordance with the similar fatty acid content analyzed between WT and KO strains. Nevertheless more experiments with other fatty acids and other conditions are needed for this conclusion.

The generation of strains with the *DES*22A-eGFP and *DES*22B-eGFP gene fusion allowed to localize the proteins in the nucleus with a strong signal surrounding it, in accordance with an ER localization previously reported for a fatty acid Δ12 desaturase from the same ciliate (44). As expected, the Des22p:eGFP fusions were significantly labeled after supplementation of the media with cholesterol. *DES22*A and *DES*22B genes are the second (75.4-fold) and third (70.1-fold) most upregulated genes in the differential genome-wide transcriptomic previously reported (24). These desaturases most probably use cytochrome *b*_5_ and cytochrome *b*_5_ reductase as ETC, since this ETC was the one reported in all other enzymes belonging to the FAHD superfamily in eukaryotic organisms localized in ER.

In the yeast complementation assay, Des22Ap and Des22Bp are likely to acquire the electrons from the yeast cytochrome b5 (Uniprot ID P40312) and the NADH-cytochrome b5 reductase (probably the Uniprot ID P38626). Previous publications have shown that heterologous expression of fatty acid desaturases from different organisms, including the D6 fatty acid desaturase of *T. thermophila*, can use this transport chain (45, 46). Heterologous expression of functional *T. thermophila* cytochrome b5–dependent C-5(6) sterol desaturase in yeast was also feasible (47). In addition, the biochemical characterization of the *T. thermophila* C-22 desaturase showed a strong requirement for the use of this ETC (15). Therefore, the similar localization pattern with Δ12 fatty acid desaturase, as well as that of the C5 sterol desaturase, which also uses cytochrome *b*_5_ and cytochrome *b*_5_ reductase (10), suggests a shared use of this ETC.

NISE enzymes are widespread in the three domains of life and appear to be originated by HGT (more often in prokaryotes) or *via* gene duplication followed by diversification (20). Phylogenetic and parametric methods have shown to be useful for inferring HGT (32). Although our analysis shows a patchy distribution of C-22 sterol desaturase orthologous genes in the tree of life, which could be a mark of HGT, the parametric analyses were unable to detect it, showing no differences in codon usage bias between the sterol C-22 desaturases and other members of FADs in different species of *Tetrahymena*. On the contrary, our evolutionary analysis showed to be compatible with a process of genetic duplication previous to functional diversification. Many studies indicate that soon after a genetic duplication event, the fixation rate of nonsynonymous substitution is increased by positive Darwinian selection or by the relaxation of selective constraints, allowing proteins to adapt to a new function (48, 49). In our analysis, the branches, soon after the duplications that will originate the stearoyl-CoA and C22 desaturase families, show a significant increase in the average rate of nonsynonymous substitutions that will later decrease showing a purifying selection in both clades, suggesting an event of gene duplication in the FAHD superfamily as the most plausible scenario for the origin of this family in ciliates.

The generation of analogous enzymes such as the ATP-grasp superfamily, HD hydrolases, and alkaline phosphatases, among others, have often been reported in prokaryotic and eukaryotic organisms (22). In fact, another event within the FAHD superfamily could be the generation of the alkane ω-hydroxylase commented above, although a more exhaustive evolutionary analysis is needed to demonstrate this hypothesis. Gene duplication in *T. thermophila* has shown to be a well-established trait, particularly among large gene families involved in sensing and responding to environmental changes (50). The FAHD is also a large gene family that has been considerably expanded in the ciliate (51) with several family members, such as the front-end desaturases, methyl-end desaturases, and stearoyl-CoA desaturases that are involved in the response to environmental changes, such as homeoviscous adaptation (44, 52). With regard to the sterol C-22 desaturase, we do not know if there is an involvement in any role related to these processes or any other physiological response. Nevertheless, the high purifying selection estimated in this work as well as the tight regulation in gene expression by sterols (24) suggest an essentially conserved biological role. In the presence of sterols, *Tetrahymena* suppresses the synthesis of tetrahymanol and replaces it by the sterol modified by the action of C-5, C-7, and C-22 desaturases. This process leads to a profound membrane lipid alteration (change in phospholipid and fatty acyl chain composition) that markedly decreases membrane fluidity with the consequent decrease in swimming velocity or even no movement at all at 15 °C (53, 54). The C-22 sterol desaturase could be involved in this process, allowing a further decrease of membrane fluidity by decreasing the side chain volume and thus increasing the condensing capacity of sterols (55). Another possible explanation for the presence and conservation of the sterol C-22 desaturases as well as the other C-5 and C-7 desaturases in an organism that neither synthesize nor require sterols could be the synthesis of steroid hormones with a hitherto unknown role. Although it has been postulated that steroid hormones strongly influence the chemotaxis in *Tetrahymena* (56), it is no clear yet, if the ciliate can store or produce steroid hormones (57).

*T. thermophila* lives in aquatic environments in the presence of exogenous sterols from different sources such as phytoplankton, higher plants, and algae from where it can assimilate and modify them. In its absence, it has the ability to synthesize tetrahymanol. The HGT origin of the genes involved in the metabolism of sterols and synthesis of hopanoids (58) and now the neofunctionalization reported in this article highlight the importance of these processes in the evolutionary history of this ciliate.

In summary, current findings complete the molecular identification of the repertoire of sterol-modifying enzymes in the ciliate whose activities were first described 50 years ago. It also stresses the widespread existence of NISE among different lineages of the tree of life as well as the suitability for the use of *T. thermophila* as a valuable model to investigate the evolutionary process of large enzyme families.

## Experimental procedures

### Strains, plasmids, and microorganism growth conditions

*S. cerevisiae* strains BY4741 (*MATa*, *his3Δ1*, *leu2Δ0*, *met15Δ0*, *ura3Δ0*) and *erg5* JA1-10B (*MATa*, *his7-2*, *trp1-289*, *ura3-52 ade5*, *erg5-1*) were retrieved from the Yeast Knockout Collection (Horizon Discovery). *S. cerevisiae* cultures were grown in YPD medium (2% proteose peptone (Britania), 1% yeast extract (Britania), 2% glucose (Cicarelli)), and YNB medium (Sigma Aldrich) supplemented with 0.02% histidine, 0.01% leucine, 0.015% lysine, 0.01% tryptophan; 0.005% adenine, and 0.02% methionine.

*T. thermophila strains* CU428.2 *mpr1-1/mpr1-1* (*MPR1*; mp-s, VII), B2086.2 (II), and CU427 *chx1-1/chx1-1* (CHX1; cy-s, VI), designated as “wildtype”, and plasmids pBS-MnB-3 and pmEGFP were retrieved from the *Tetrahymena* Stock Center (Cornell University). The pMcoDel t plasmid was a gift from K. Mochizuki (Institute of Human Genetics, CNRS). The GFP-tagged Δ12 fatty acid strain (Δ12 FAD-GFP) was previously generated by our group (44). *T. thermophila* cultures were grown in PPYE medium (1% proteose peptone (Britania), 0.1% yeast extract (Britania), 0.5% glucose (Britania), and 0.003% iron (III) citrate (Sigma-Aldrich)) at 30 °C and 80 rpm in 250 ml Erlenmeyer flasks. Paromomycin (Sigma-Aldrich) was added from a 200 mg/ml PPYE stock solution when indicated, along with 1 μg/ml of CdCl_2_, prepared as a 1 mg/ml stock solution in water. A 1:25 dilution of a 24-h culture was inoculated daily into the cultures. For sterol supplementation, 20 μg/ml of pro-vitamin D_3_ (5,7-cholestadien-3β-ol), cholesterol (5-cholesten-3β-ol), or lathosterol (7-cholesten-3β-ol) were added from 5 mg/ml stock solution prepared in absolute ethanol. Sterols used in this work were acquired in Steraloids Inc. Cells were counted in a Neubauer chamber.

### Standard DNA and RNA manipulation procedures for PCR and RT-PCR

Genomic DNA from *T. thermophila* CU428 (WT strain) was prepared as previously described (59). Isolation of plasmid DNA from *E. coli* was performed using a QIAprep Spin Miniprep DNA purification system kit (Qiagen). Total RNA was prepared from *T. thermophila* cultures using TRIzol reagent (Invitrogen). Nucleic acid fragments were amplified by PCR using FIREPol DNA polymerase (Solis Biodyn) or KAPA HiFi DNA Polymerase (Kapa Biosystems) when high-fidelity amplifications were needed. Moloney murine leukemia virus reverse transcriptase (Promega) was used to conduct reverse transcription reactions following the manufacturer’s instructions.

### Functional characterization of the *T. thermophila* desaturases in yeast

Codon-optimized versions of *DES22A* and *DES22B* were synthesized (Genscript) for expression in *S. cerevisiae. DES22A* was cloned in the p426GPD vector and *DES22B* in the pYES2 vector. A strain of *S. cerevisiae*, which does not exhibit endogenous C22 sterol desaturase activity (*erg5*), was transformed with the *DES22A* and *DES22B* expression constructs. Cultures of *S. cerevisiae* were inoculated into selective medium, incubated overnight at 30 °C, and then transferred to 40 ml of YNB medium containing 2% w/v glucose. Cultures of p426GPD-DES22A were grown at 30 °C for 48 h and then harvested for lipid extraction. Cultures of pYES2-DES22B were grown at 30 °C for 72 h, and then cells were centrifuged, washed with distilled water, and resuspended in YNB medium containing 1% galactose. Cells were harvested after 24 h induction. No differences in growth were observed between the recombinant yeast strains.

### Construction of DES22 mutants in *T. thermophila*

For *DES22A* and *DES22B* gene disruption by somatic knockout, the transformation sequences C9290KO and C5010KO were constructed. The 730 bp upstream and 790 bp downstream TTHERM_00129290 (I7M1E1) flanking regions and 810 bp upstream and 820 bp downstream TTHERM_00085010 (Q236U7) flanking regions were synthesized separately (Genscript). For each construct, both upstream and downstream sequences were cloned into plasmid pBS-MnB-3 at XhoI/SalI and EcoRI/NotI sites. The constructs were then released with XhoI/NotI RE and introduced into the macronucleus of *T. thermophila* CU428.2 cells by biolistic transformation (59). Transformants were recovered in 50 ml SPP medium containing 1.0 μg/ml CdCl_2_, selected upon growing in 120 μg/ml paromomycin, and subsequently transferred to PPYE medium with increasing concentrations of paromomycin until no further growth could be obtained (∼70 mg/ml). For the generation of *T. thermophila* double knockout cells, the *DES22A* gene was eliminated by a targeted ectopic DNA elimination (codeletion) procedure and the *DES22B* gene by a somatic knockout procedure. For the codeletion procedure, a 1.03 kb sequence of the TTHERM_00129290 CDS was amplified and cloned into the NotI site of the pMcoDel plasmid and introduced into conjugating wildtype cells (CU428.2/B2086.2) at the macronucleous early developmental stage (7 h) by electroporation. Transformants were subsequently cultured in 10 mM Tris (pH. 7.5) overnight and for an additional 3 h in PPYE medium. Cells possessing pMcoDel were selected using 100 μg/ml paromomycin in PPYE medium. Progeny cell lines showing higher coDel efficiency were subsequently selected, and five clones from each cell line were isolated, and their target locus was analyzed by genomic PCR (60). The DES22A KO strain was subsequently transformed by a somatic knockout procedure with the C5010KO construct and selected with high concentration of paromomycin (4 mg/ml). Several clones were subsequently grown in PPYE medium with increasing concentrations of paromomycin until only the KO loci of both genes were amplified by genomic PCR (∼45 mg/ml). For localization of Des22Ap and Des22Bp, monomeric enhanced GFP (mEGFP) was fused at the C terminus of both genes *via* homologous recombination using transformation sequences C9290GFP and C5010GFP. C-terminal fragments of 823 and 817 bp of each gene (Up) were amplified by PCR and cloned into pmEGFP at *Sac*I/*Bam*HI sites. A downstream flanking region of 818 and 918 bp of each gene were amplified and cloned into pmEGFP+Up at *Xho*I and *Kpn*I sites. The constructs were then released with *Sac*I and *Kpn*I restriction enzymes and introduced into the macronucleus of *T. thermophila* CU428.2 cells by biolistic transformation. Detailed sequences synthesized are listed in Table S1.

### Competitive assays for C22 desaturase activity

Assays were performed with microsomes of WT *T. thermophila* cells cultivated with 20 μg/ml of cholesterol. Microsomes were obtained as indicated in Nusblat *et al.* (15). The incubation mixture contained 2 ml of sample of microsomes at 1 mg/ml of protein containing [1,2^3^H] CHOL (3 mCi/ml, 60 nM, final concentration). The reaction was started by adding 1 mM NADH under gentle stirring at 30 °C for 3 h under an oxygen atmosphere. The nonradiolabeled substrates were added to a final concentration up to 50 mM. The reaction was stopped by adding 1 ml of 2 M NaOH and heated at 60 °C during 1 h.

### Sterol extraction and identification

*S. cerevisiae* cells from cultures were collected by centrifugation at 3000*g* for 5 min at 4 °C and washed twice with 20 ml of distilled water, and the lipids were extracted according to (61). The organic phase was evaporated to dryness under a N_2_ stream, and the lipids were saponified. After 2-fold extraction with 2 ml hexane, the organic solvent was evaporated under N2 stream, and the residue was resuspended in 50 ml of distilled pyridine. One hundred microliters of acetic anhydride were added, and the mixture was incubated for 40 min at 80 °C. The composition of stearyl acetate ester derivatives was analyzed by running samples through an SPB-1 column (30m_0.25mm_0.25 mm; Supelco) in a Shimadzu GC-2010 Plus gas chromatograph. The column was temperature programmed at 5 °C/min from 160 to 320 °C and subsequently held for 10 min at 320 °C. MS was carried out using a GCMS-QP2010 Plus mass detector and operated at an ionization voltage of 70 eV with a scan range of 20 to 600 atomic mass units. The retention times and mass spectra of all new peaks obtained were compared with those of standards (Sigma-Aldrich) and those available in the National Institute of Standards and Technology mass spectral library and with the compounds published in (62).

Samples of 2 ml of *T. thermophila* cells from cultures supplemented with sterols were collected by centrifugation (3000*g*, 5 min at 4 °C), washed, resuspended in water, and submitted to lipid saponification by the addition of 1 volume of 2 M NaOH in methanol-water (1:1, vol/vol) at 60 °C for 1 h to get the free alcohol sterols. After being cooled, lipids were extracted according to the Bligh and Dyer method (61) and separated by high-performance liquid chromatography on a C18 Ultrasphere column, using methanol/water (98:2, vol/vol) as the mobile phase at 41 °C. The absorbency of the eluates was monitored at 210 nm (quantification and identification) and 285 nm (identification). An absorbency in the latter is indicative of the formation of conjugated Δ5,7-dienes. The identity of each compound was established previously (15, 63). Stigmasterol was used as internal standard and added prior to extraction at a final concentration of 20 μg/ml. The bioconversion of radiolabeled cholesterol were analyzed by HPLC coupled to a Flo-one Beta Radio 105 chromatography detector, following the manufacturer’s instructions (Radiomatic, Canberra Company Q8) (15).

### Fatty acid analysis

*T. thermophila* WT and KO cells from cultures grown at 30 °C were collected by centrifugation at 3000*g* for 2 min at 4 °C and washed twice with distilled water, and total lipids were extracted by Bligh and Dyer method. The composition in fatty acids were obtained by the generation of methyl ester (FAME) derivatives and analyzed by GC-MS in a Hewlett Packard HP 6890 gas chromatograph equipped with a Zebron ZB-5 column following the procedure descripted in (44).

### Microscopic analysis

*Tetrahymena* cells expressing Des22Ap-GFP and Des22Bp-GFP were fixed with 2% paraformaldehyde in 50 mM Hepes, pH 7.0, for 10 min at room temperature and permeabilized with ice-cold 0.1% Triton X-100 in 50 mM Hepes pH 7.0 for 10 min. After washing the fixed cells three times with ice-cold Hepes, they were treated with a blocking solution (1% bovine serum albumin in TBS buffer) at room temperature for 1 h and incubated in blocking solution containing anti-GFP primary antibody (A-11122; Invitrogen) for 30 min at a 1:400 dilution. Cells were washed three times with TBS buffer containing 0.1% BSA for 5 min each and subsequently incubated at room temperature donkey anti-rabbit-Alexa Fluor 488 secondary antibody (A21206; Invitrogen) at a 1:200 dilution in blocking solution for 1 h at a 1:200 dilution. After one wash in TBS buffer containing 0.1% BSA, cells were washed twice with 50 mM Hepes pH 7.0 and mounted with anti-bleaching solution (0.2% n-propyl gallate, 90% glycerol in PBS). Digital images were collected using a Nikon Eclipse TI-S L100 fluorescence microscope. Images were analyzed with Image J 1.50i (Wayne Rasband, National Institute of Health).

### Bioinformatics and phylogenetic analyses

A dataset comprising C22 sterol desaturases was generated by homologous sequence searches using the I7M1E1 and Q236U7 proteins from *T. thermophila* as queries. The OMA (Orthologous MAtrix) database was used to retrieve reliable sequences (64). To retrieve homologous sequences from ciliates, the databases of *E. octocarinatus*, *I. multifiliis* (http://ich.ciliate.org), *O. trifallax* (http://oxy.ciliate.org), *S. lemnae* (http://stylo.ciliate.org), *T. thermophila* (http://ciliate.org), *Stentor coeruleus* (http://stentor.ciliate.org), *P. tetraurelia* (http://paramecium.cgm.cnrs-gif.fr/), and *P. persalinus* (http://ciliates.ihb.ac.cn/database/species/pp) were used. All the sequences were scanned for the tripartite conserved motif (H-x (3,4)- H-x(3,150)- H-x (2,3)- H-H- x (50,250)-[HQ]-x (2,3)-H-H) using the ScanProsite tool, and those not matching were discarded. Sequences of other FAD families were retrieved from the Universal Protein Resource (UniProt) databases using representative prokaryotic and eukaryotic FADs from the Swiss-Prot database as queries. Only the sequences with confirmed activity were used (65). Redundant sequences were excluded from the dataset sequences using CD-HIT with a sequence identity cut-off of 0.99 (66) (Li, 2006). FAD CDS and protein sequences from *Tetrahymena* species (*T. thermophila*, *Tetrahymena malaccensis*, *T. elliotti, Tetrahymena pyriformis*, *Tetrahymena vorax*, *T. borealis*, *Tetrahymena canadensis*, *Tetrahymena empidokyrea*, *Tetrahymena shanghaiensis*, and *Tetrahymena paravorax*) were retrieved from the *Tetrahymena* comparative genome database (67) using BLAST of *Tetrahymena thermophila* sequences as queries, text search, and by PFAM motive search (PF00487). The sequences used for the bioinformatic analyses are listed in Table S2. Amino acid sequences were aligned using the MAFFT algorithm (68) implemented in the online resource at CBRC, Japan, using the E-INS-i Iterative refinement method, which showed to be the most accurate for the FAD sequences (65). Phylogenetic relationships were determined by the maximum likelihood method using PhyML 3.0 optimized by subtree pruning and regrafting and nearest neighbour interchange with a support of 100 bootstraps (69) at the ATGC online resource. The evolutionary models for the multiple sequence alignment were selected using the smart model selection method (70). For estimation of the selective pressure in different branches of the *Tetrahymena* FAD families, the nonsynonymous (dN) to synonymous substitution (dS) rate ratio ω (=dN/dS) was calculated with CODEML analysis from the PAML package, using free-ratios of the branch model (model = 2, NSsites = 0) (71, 72). CUB analysis was performed using the CodonW package (http://codonw.sourceforge.net) and custom scripts. The ENc-GC3s plot and principal component analysis were used to analyze codon usage variation between FADs (73, 74). The parity rule 2 analysis was performed in order to evaluate the impact of mutation and selection on CUB, and the neutrality plot was used to measure the degree of relative neutrality when selection pressure plays a prime role in evolution (75).

## Data availability

All data and supplemental information are available in the manuscript.

## Supporting information

This article contains supporting information (69, 70, 81).

## Conflict of interest

A. D. N., A. D. U., and C. B. N. are members of the Consejo Nacional de Investigaciones Científicas y Técnicas. The authors declare that they have no conflicts of interest with the contents of this article.
