# Supplementary material for: A novel Tetrahymena thermophila sterol C-22 desaturase belongs to the fatty acid hydroxylase/desaturase superfamily
Source: J Biol Chem. 2022 Aug 18;298(10):102397. doi: 10.1016/j.jbc.2022.102397 (PMC9485055; doi:10.1016/j.jbc.2022.102397)
Supplement: Supplemental Figures S1–S4 [file mmc1.docx]

SUPPORTING INFORMATION:

**Identification and classification of a novel *Tetrahymena thermophila* sterol C-22 desaturase**

María L Sanchez Granel^a,1^, Nicolás G Siburu^b,1^, Annamária Fricska^a^, Lucas L. Maldonado^c^, Laura B. Gargiulo^a^, Clara B. Nudel^a^, Antonio D. Uttaro^b,2^ and Alejandro D. Nusblat^a,2^

a Instituto de Nanobiotecnología (NANOBIOTEC), CONICET, Facultad de Farmacia y Bioquímica, Universidad de Buenos Aires, Junín 956, C1113AAD, Buenos Aires, Argentina.

b Instituto de Biología Molecular y Celular de Rosario, CONICET, Facultad de Ciencias Bioquímicas y Farmacéuticas, Universidad Nacional de Rosario, Ocampo y Esmeralda s/n, S2000FHQ, Rosario, Argentina.

c Instituto de Investigaciones en Microbiología y Parasitología Médica (IMPaM), CONICET, Facultad de Medicina, Universidad de Buenos Aires, Junín 956, C1113AAD, Buenos Aires, Argentina.

1 Both authors contributed equally to this work.


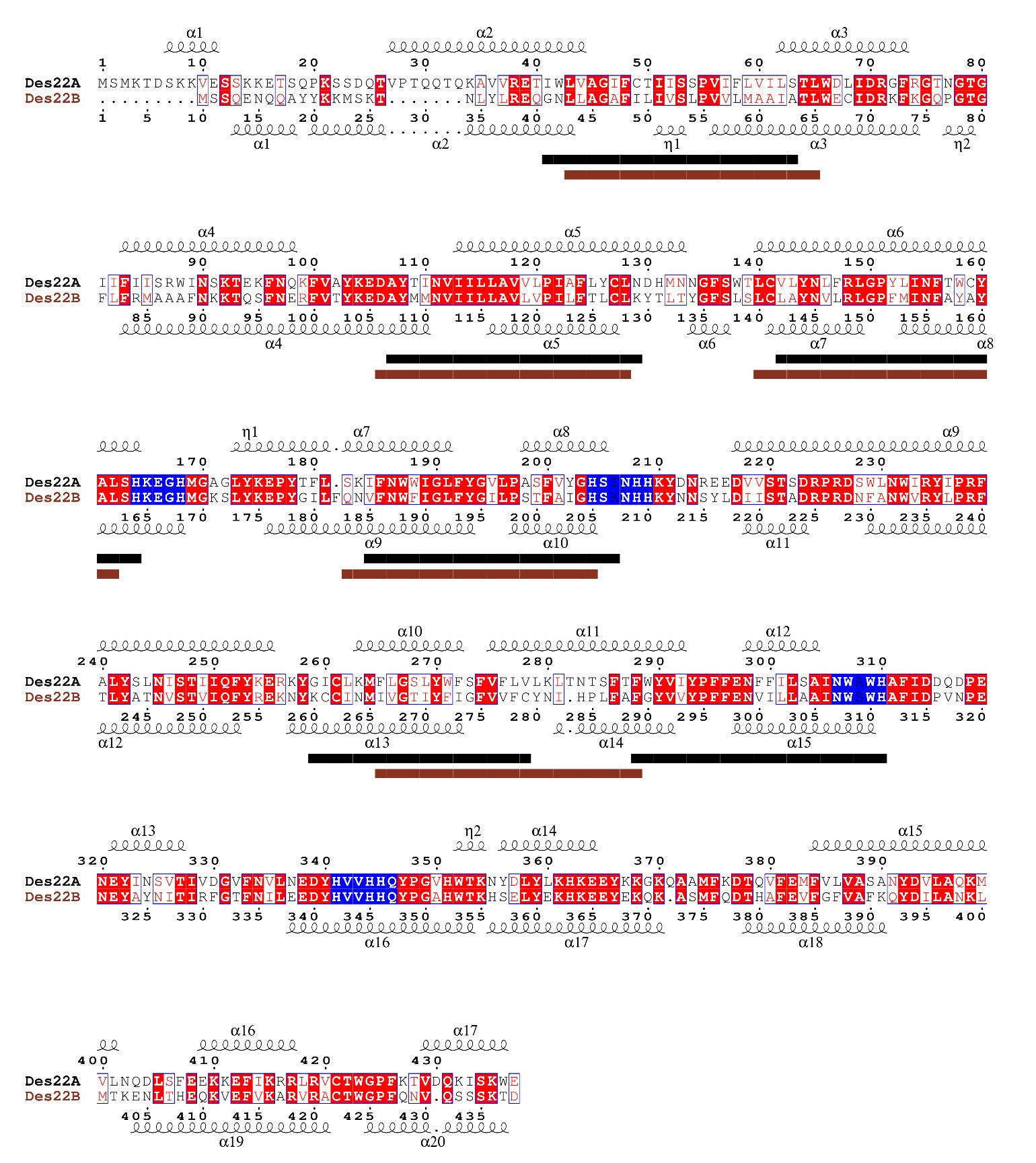


**Figure S1.** Sequence similarities and secondary structure of Des22 sterol desaturases of *T. thermophila.*

Histidine clusters are indicated in blue, predicted transmembrane regions are indicated with solid black and brown rectangles and α-helices are rendered as squiggles. The figure was generated using the Espript program (81).


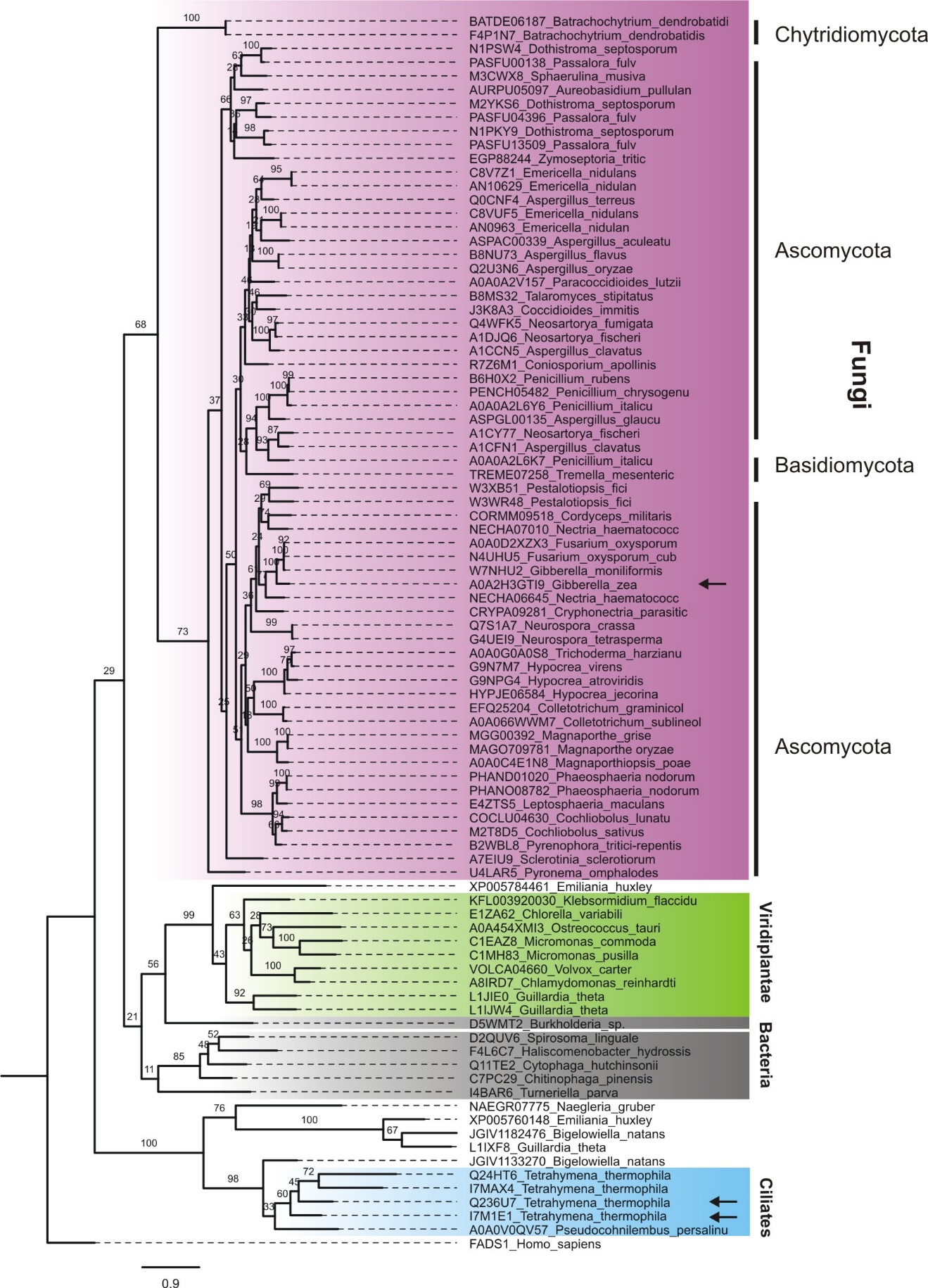


**Figure S2.** Phylogenetic analysis of C22 sterol desaturase. For the phylogenetic analysis, the MAFFT program with the E-INS-i iterative refinement method was used to obtain the multiple alignment from 90 protein sequences listed in Table S2. This MSA was used to construct the phylogeny by the maximum- likelihood method using PhyML 3.0 optimized by SPR and NNI and a support of 100 bootstraps (69). The model LG+G+I for the MSA was selected using the Smart Model Selection method (SMS) (70). Black arrows indicate the acylamide-delta-3(E)-desaturase from the fungus *Gibberella zeae* and the C22-sterol desaturases from *Tetrahymena thermophila*.


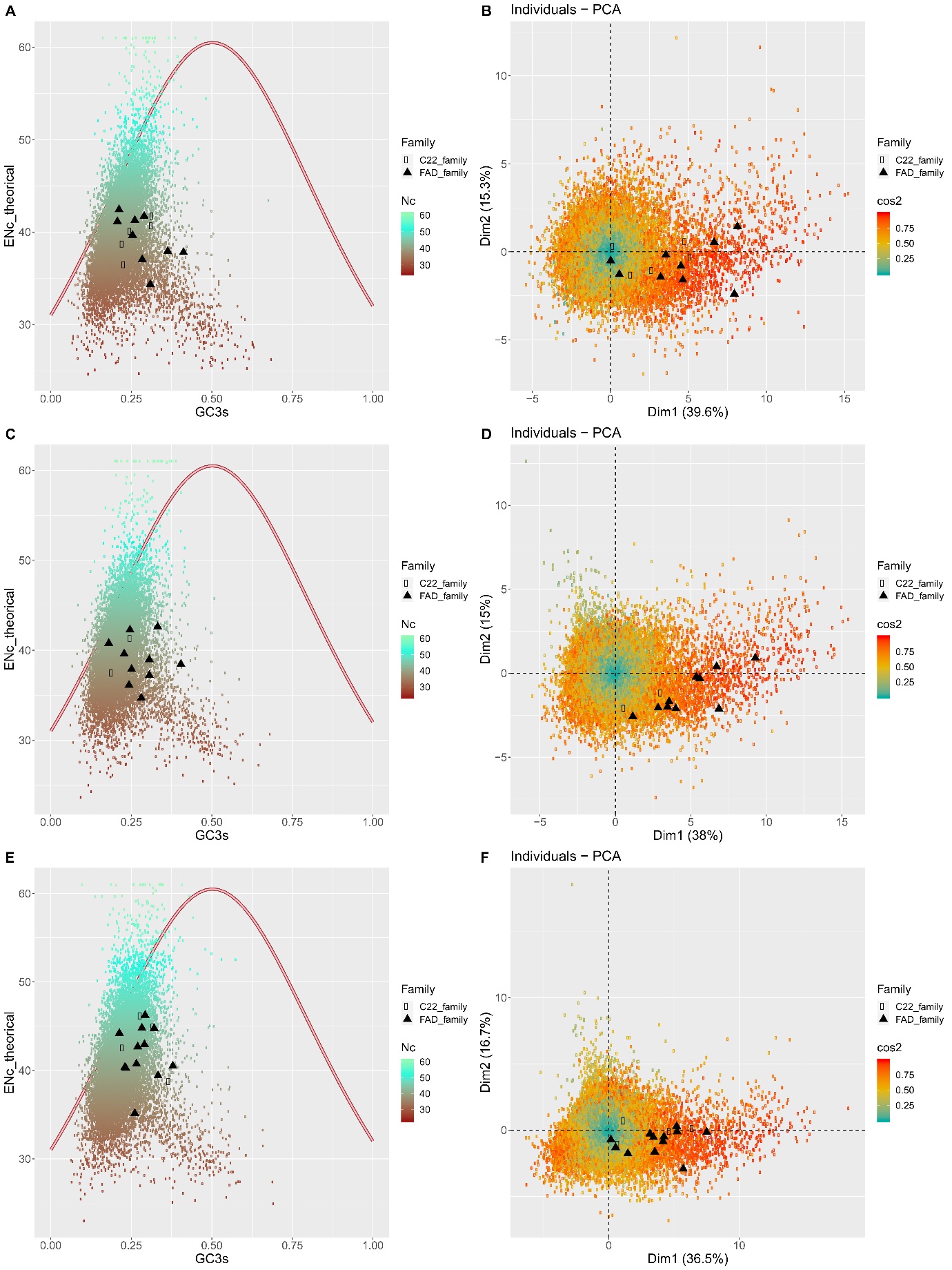


**Figure S3.** Distribution of the effective number of codons (ENc) in relation to the GC3 content (GC content of synonymous codons in the third position) of T. *borealis, elliotti* and *malacensis* genes (A, C and E respectively). The solid line represents the expected curve when codon usage bias is only affected by mutation pressure (up). Principal component analysis of relative synonymous codon usage in *T. borealis, T. elliotti* and *T.* *malacensis* (B, D and F respectively)*.* The axis shows the percentage accounting for the total RSCU variation (down). Black circles and triangles indicate genes belonging to the C22 sterol desaturase family and to other members of the fatty acid desaturase superfamily respectively.


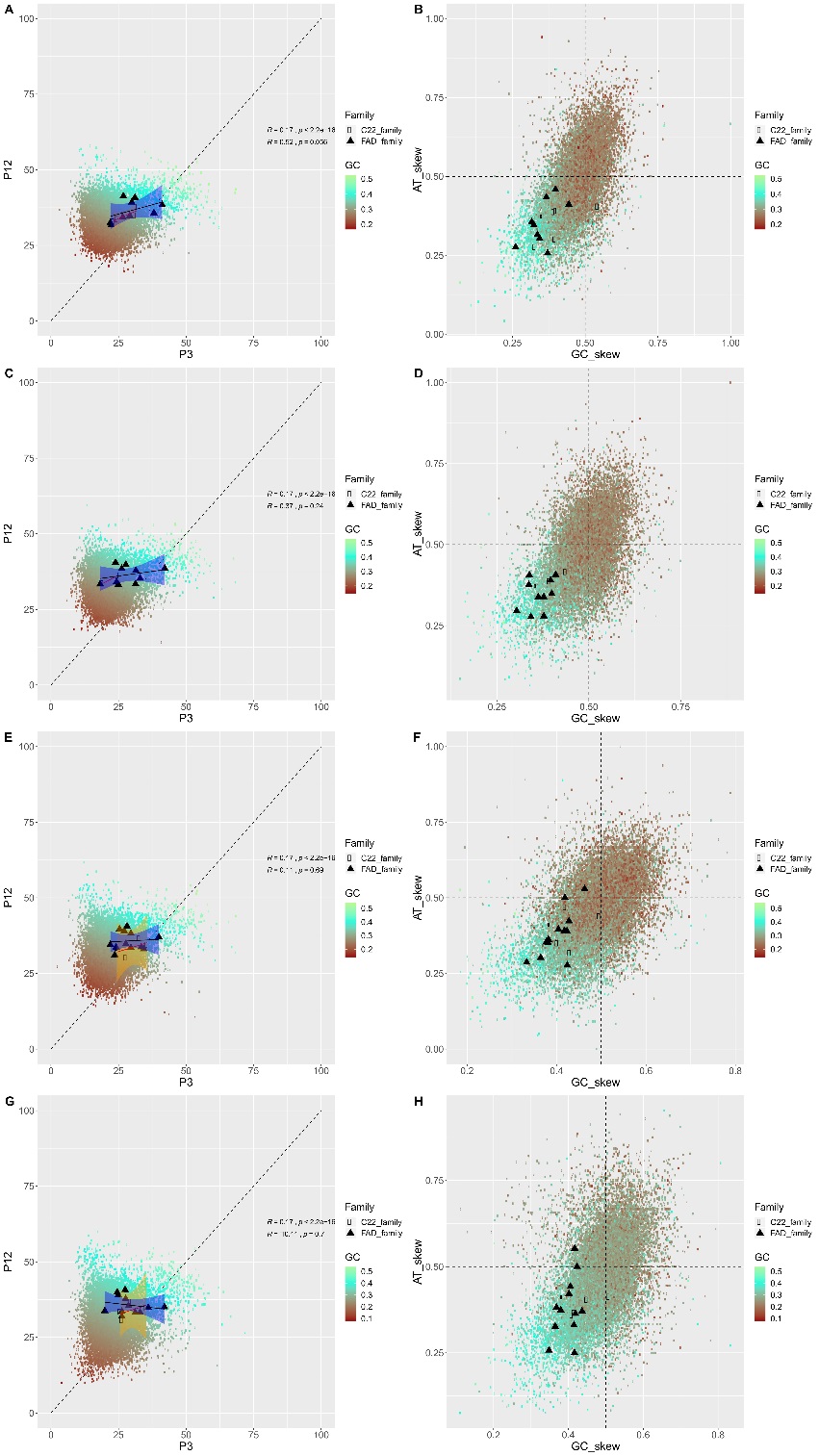


**Figure S4.** The Parity rule 2 plot analysis evaluates the impact of mutation and selection on CUB by the variability of the DNA G + C content. Deviations are analyzed in terms of an excess of the number of guanines relative to cytosines or adenines relative to thymines and the bias is measured by GC and AT skews, (G)/(G+C) and (A)/(A+T), respectively. If the codon usage in genes is determined only by a mutational bias, the G+C and A+T should be used proportionally in all synonymous codons. The C22 gene distribution towards the lower left quadrant (C and T are more frequently used than G and A) in the four *Tetrahymena* species provides additional evidence that other factors in addition to mutational bias contribute to shaping the codon usage in this family. Genes of *T. borealis, T. elliotti, T. malacensis* and *T. thermophila* are shown in AB, CD, EF and GH respectively.
