# Supplementary material for: A novel Tetrahymena thermophila sterol C-22 desaturase belongs to the fatty acid hydroxylase/desaturase superfamily
Source: J Biol Chem. 2022 Aug 18;298(10):102397. doi: 10.1016/j.jbc.2022.102397 (PMC9485055; doi:10.1016/j.jbc.2022.102397)
Supplement: Table S3 [file mmc4.docx]

**Table S3. Fatty acid composition in WT and KODes22 strains.**

| **Species** | **WT** | | | | **Des22A KO** | | | | **Des22B KO** | | | | **Double KO** | | |
| --- | --- | --- | --- | --- | --- | --- | --- | --- | --- | --- | --- | --- | --- | --- | --- |
| 14:0 | 1,53 | ± | 0,78 | 2,92 | | ± | 0,62 | 3,87 | | ± | 0,51 | 1,48 | | ± | 0,40 |
| 15:0 | 1,60 | ± | 0,57 | 1,78 | | ± | 0,47 | 1,54 | | ± | 0,68 | 2,34 | | ± | 0,49 |
| 16:0 | 12,70 | ± | 0,72 | 11,39 | | ± | 1,74 | 13,95 | | ± | 0,53 | 11,96 | | ± | 0,65 |
| 16:1 (9) | 4,55 | ± | 1,05 | 6,78 | | ± | 0,49 | 4,97 | | ± | 1,20 | 6,37 | | ± | 0,24 |
| 17:0 | 5,47 | ± | 0,88 | 5,21 | | ± | 0,55 | 4,32 | | ± | 0,60 | 4,25 | | ± | 1,58 |
| 18:0 | 4,51 | ± | 0,49 | 5,33 | | ± | 0,65 | 6,24 | | ± | 0,75 | 5,04 | | ± | 0,45 |
| 18:1 (9) | 9,38 | ± | 1,08 | 11,29 | | ± | 0,94 | 9,93 | | ± | 0,42 | 11,46 | | ± | 0,86 |
| 18:1 (11) | 5,09 | ± | 0,78 | 4,55 | | ± | 0,46 | 6,58 | | ± | 0,58 | 3,87 | | ± | 1,21 |
| 18:2 (9,12) | 12,04 | ± | 0,95 | 12,32 | | ± | 1,02 | 13,56 | | ± | 1,87 | 14,39 | | ± | 0,73 |
| 18:2 (11,13) | 2,54 | ± | 0,92 | 0,97 | | ± | 0,53 | 1,61 | | ± | 0,53 | 1,85 | | ± | 0,70 |
| 18:3 (6,9,12) | 34,20 | ± | 0,69^ab^ | 30,99 | | ± | 1,49 | 27,66 | | ± | 1,53^a^ | 28,99 | | ± | 1,82^b^ |
| 18:0 (2(OH)) | 2,25 | ± | 0,70 | 1,88 | | ± | 0,76 | 2,17 | | ± | 0,94 | 2,12 | | ± | 0,64 |
| Total SFA | 28,06 | | | | 28,51 | | | | 32,09 | | | | 27,20 | | |
| Total MUFAs | 19,02 | | | | 22,62 | | | | 21,48 | | | | 21,70 | | |
| Total PUFAs | 48,78^ab^ | | | | 44,28^a^ | | | | 42,83^b^ | | | | 45,24 | | |

WT and KODes22 cells were grown at 30ºC, and fatty acid composition was evaluated as described under Materials and Methods. Results are shown as the mean ± SD of 3 independent cultures. Two- way ANOVA followed by Bonferroni’s multiple comparison test was used to assess differences in fatty acid profiles of WT and KO strains. Means in the same row with different superscripts are significantly different (*P*< 0.001) respect to the value recorded in WT cells
